# Comparison of Missing Data Infilling Mechanisms for Recovering a Real-World Single Station Streamflow Observation

**DOI:** 10.3390/ijerph18168375

**Published:** 2021-08-07

**Authors:** Thelma Dede Baddoo, Zhijia Li, Samuel Nii Odai, Kenneth Rodolphe Chabi Boni, Isaac Kwesi Nooni, Samuel Ato Andam-Akorful

**Affiliations:** 1 Binjiang College, Nanjing University of Information Science & Technology, No.333 Xishan Road, Wuxi 214105, China; nooni25593@alumni.itc.nl; 2 College of Hydrology and Water Resources, Hohai University, Nanjing 210098, China; zjli@hhu.edu.cn; 3 Office of the Vice Chancellor, Accra Technical University, Accra GA000, Ghana; snodai@yahoo.com; 4 College of Computer and Information Engineering, Hohai University, Nanjing 211100, China; boni_kenneth@yahoo.fr; 5 Wuxi Institute of Technology, Nanjing University of Information Science & Technology, Wuxi 214105, China; 6 School of Geographical Sciences, Nanjing University of Information Science & Technology, Nanjing 210044, China; 7 Department of Geomatic Engineering, Kwame Nkrumah University of Science and Technology, Kumasi AK000, Ghana; aakorful@gmail.com

**Keywords:** missing data, univariate imputation, multiple imputation, SPSS, R, China

## Abstract

Reconstructing missing streamflow data can be challenging when additional data are not available, and missing data imputation of real-world datasets to investigate how to ascertain the accuracy of imputation algorithms for these datasets are lacking. This study investigated the necessary complexity of missing data reconstruction schemes to obtain the relevant results for a real-world single station streamflow observation to facilitate its further use. This investigation was implemented by applying different missing data mechanisms spanning from univariate algorithms to multiple imputation methods accustomed to multivariate data taking time as an explicit variable. The performance accuracy of these schemes was assessed using the total error measurement (TEM) and a recommended localized error measurement (LEM) in this study. The results show that univariate missing value algorithms, which are specially developed to handle univariate time series, provide satisfactory results, but the ones which provide the best results are usually time and computationally intensive. Also, multiple imputation algorithms which consider the surrounding observed values and/or which can understand the characteristics of the data provide similar results to the univariate missing data algorithms and, in some cases, perform better without the added time and computational downsides when time is taken as an explicit variable. Furthermore, the LEM would be especially useful when the missing data are in specific portions of the dataset or where very large gaps of ‘missingness’ occur. Finally, proper handling of missing values of real-world hydroclimatic datasets depends on imputing and extensive study of the particular dataset to be imputed.

## 1. Introduction

Missing data are an issue, whether we like it or not [1]. For instance, incomplete streamflow data (missingness) are faced frequently in practice due to instrument failure or damage, and a host of other factors [1,2,3]. Missing values in data can pose complications, due to the fact that further data processing and analysis mostly require complete datasets [4]. If not properly handled, they may introduce bias into the observed datasets, which subsequently lead to uncertainty in hydrological model outputs (particularly in cases where the data span or length is limited) [5]. Thus, properly filling streamflow missing data would not only improve model evaluation but facilitate water resource conservation efforts in water-limited regions across the world.

Missing data are not problematic per se; but rather it is the handling that raises conceptual difficulties and computational challenges [6]. Selecting a particular method is based on several factors, including the number of missing observations, seasonal characteristics of missing observations, and available data from neighboring stations [7].

A runoff-gauging system in a watershed provides hydrologic time series data needed to plan, develop, implement, and manage water resources projects. Long periods of historical records of data not only enhance our understanding of changes in water resources both in space and time but also improve our skills in hydrological modelling, particularly in a changing climate [8]. Therefore, missingness in streamflow datasets must be refilled with appropriate values. In statistics, this process is called imputation [4].

According to Chandrasekaran et al. [9], observed datasets may mostly be univariate, or multivariate datasets may lack correlation. A univariate time series is a sequence of single observations at consecutive points in time, and even though generally considered as one column of observations, time is actually an implicit variable [4]. Also, Moritz et al. [4] stated that imputation of univariate time series are a special challenge and Moritz and Bartz-Beielstein [10] developed algorithms for missing value imputation of univariate time series in R, based on the input data characteristics such as seasonality, or trend, or both.

Multiple imputation is acclaimed as an appropriate way of handling incomplete data, since it considers the uncertainty in the imputations [11]. Multiple imputation (MI) [12], Expectation-Maximization (EM) [13] and other algorithms of missing data imputation have become very popular in recent studies, however, they require multivariate data because they depend on inter-attribute or inter-variable correlations to estimate values for the missing data. Moreover, since univariate time series have no other correlated variables except time, these algorithms fail for missing data imputation of univariate time series [4,9].

Different methods are used for the infilling or reconstruction of missing data, including: univariate time series [9,14,15,16,17,18], hydroclimatic values, and streamflow values [7,19,20,21,22,23,24,25,26,27], among others. However, some drawbacks from some of these studies have been identified. Some of these studies (especially for univariate time series) focus on the introduction of somewhat novel complex algorithms, which are supposed to perform satisfactorily across board for all univariate time series without much attention to the fact that different data possess different attributes (see [17,18,28,29,30,31,32,33]).

The other drawback is the requirements of multiple variable input data from other sources [7,26,34], which might pose a challenge when climate variables are not readily available or only data from a single streamflow outlet station is available. Many more additional drawbacks are related to the procedure used in the data processing such as hydrological models [27,35] or machine learning models (e.g., artificial neural networks (ANNs)) [2,5,7,22,24,36,37].

Despite their advantages in improving the understanding of missing data imputation, several of these studies focusing on missing data imputation methods make use of complete datasets and the application of randomly introducing different proportions of missingness to this complete data by one mechanism or another. Therefore, missing data imputation of real-world datasets to investigate how to ascertain the accuracy of imputation algorithms for these datasets are lacking, and the mechanisms of randomly introducing missing data into complete datasets may or may not properly replicate the observed field situations. In their work, Moritz and Bartz-Beielstein [10] stated that of all the available imputation methods, no specific overall best mechanism could be established. Therefore, missing data imputation involves the imputer providing efficient methods for reconstructing the missing values, especially for real-world datasets without added complexity.

The time aspect of a univariate time series dataset, although considered implicit, is a variable nonetheless. Therefore, in this study, we investigate the imputation of univariate time series with multivariate algorithms by taking time as an explicit variable and comparing their performances to the well-known univariate missing value imputation algorithms. We also apply a multivariate algorithm that can use the time characteristics of the dataset to generate and add covariates in the form of polynomials of time to the model to be able to apply multivariate imputation.

This paper contributes to our understanding and highlights recent developments in filling missing data, particularly in geographical areas that often suffer from inadequate hydrological data by tackling questions such as: (1) can multiple imputation methods be used with accuracy on univariate time series data, and how do they compare with univariate time series algorithms; and (2) how do we handle missing data in real-world data, and is only imputation enough?

This study investigated the performance of differing missing value imputation mechanisms on real-world field data with missing data whose actual values are unknown, with the aid of a reference complete dataset of the same period from another catchment.

We used approaches that take as input streamflow data from a single streamflow station to reconstruct missing streamflow data values in the Zhidan catchment in Northern China. Based on several missing data reconstruction algorithms, it is easy to use graphical user interface (GUI) statistical software to perform more complex programming, to investigate the complexity necessary to deal with missing data of univariate time series. As the streamflow data of the Zhidan catchment is field data from observation and the actual missing values are unknown, we used a similar single station observed streamflow data from the Maduwang catchment, also in Northern China, as the reference dataset and compared the results. According to van Buuren [1], imputation should not be considered as prediction, where the focus is to find the missing data algorithms which produce values closest to the true data (in cases where studies are begun with complete datasets, missing data are introduced and then imputation algorithms applied) and therefore in this study, we concentrated rather on the bias obtained from the differing missing data algorithms, and in this paper we suggest the application of other statistical metrics to be considered in the cases of real-world situations where the complete datasets are not available.

The rest of the paper is organized as follows. In Section 2, the data used and methods of study are described. In Section 3, the results are presented, and in Section 4, the results obtained from the study are discussed. The main conclusions of the study are summarized in Section 5.

## 2. Materials and Methods

### 2.1. Study Areas and Data Used

The study was conducted in the Zhidan watershed, geographically located in Shaanxi Province, China (on latitude 36°49′ N, longitude 108°46′ E) [38]. The watershed (a subset of the Yellow River watershed) is about 774 km^2^ [39] and includes an 81.3 km of main channel length with an average elevation of approximately 1230 m [38]. The area is a mountainous catchment with scant vegetation. The land use of the Zhidan watershed consists mostly of grasslands, meadows, and minor farmlands, due to the constant erosion.

**Figure 1:**
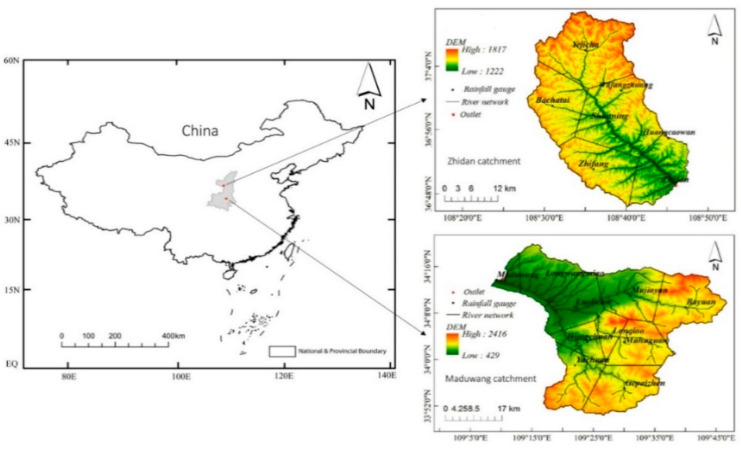
Zhidan and Maduwang catchment areas showing the river network with the meteorological stations and the hydrometeorological (outlet) stations.

The soil texture of the catchment consists of loess soil, silt soil, and saline soil and suffers from severe soil erosion [38,40]. The area lies in the middle temperate arid region and experiences continental monsoon climate according to the Köppen–Geiger climate and Kan [40]. The Zhidan watershed has six meteorological stations and one hydrometeorological (outlet) located downstream of the river (Figure 1).

The Maduwang catchment is located in the west of Shaanxi Province in Northern China [41]. The outlet station is located at 34°13′29″ N, 109°08′42″ E on the Ba River in Shaanxi Province in Northern China [39,41,42]. The catchment area above the station is approximately 1601 km^2^ with the main channel length of 30 km and an average catchment elevation of roughly 1166 m [39,41].

The catchment has a warm temperate semi-humid continental monsoon climate with rainstorms mostly concentrated in the middle of the basin [41]. The annual mean evaporation of the Maduwang catchment is 776 mm, and the annual average precipitation is 630.9 mm [41].

The mountainous areas in the catchment are steep, the valleys are vertical and horizontal, and the peaks and ridges are continuous in Qinling Mountains, with better vegetation [42], and therefore the land use of this catchment consists of evergreen forests, grasslands, and farmlands. The Maduwang watershed is also depicted in Figure 1.

We obtained daily point streamflow data from the years 2000 to 2010 from the local hydrological bureau of the Zhidan watershed. The streamflow dataset of the Zhidan watershed is a single station field dataset with missing data without knowledge of the actual values of the missing data and, therefore, can be classified as a univariate time series dataset.

Additionally, we obtained daily streamflow dataset (throughout 2000 to 2010) from the local hydrological bureau of the Maduwang watershed (a semi-humid watershed located on the Ba River in Shaanxi Province of northern China) [39,41]. The Maduwang watershed dataset was complete with no missing values.

The reason for selecting of the Maduwang watershed as the control was based on data availability and the fact that the Zhidan and Maduwang watersheds possess a similar seasonal rainfall pattern that is intense during flood seasons, with heavy rainstorms being the considerable cause of flash floods [39]. This shared seasonality is also observed in the streamflow data of these catchments. In addition, we chose the Zhidan and Maduwang catchments for this study because of lack of connective data from other catchments. The study focused on reconstructing streamflow missing data in the Zhidan watershed due to its data scarcity and lack of research in this area also pertaining to its semi-arid nature. We selected the Maduwang catchment as the appropriate control dataset to compare the missing data reconstruction algorithms due to similarities in the seasonality and distribution of their data to avoid bias in the results obtained.

To ensure the efficacy of the missing data values imputation, we also obtained the yearly flood event data of both watersheds from 2000 to 2010 to inspect for outliers in the datasets.

### 2.2. Methods

#### 2.2.1. Exploratory Data Analysis (EDA)

Exploratory data analysis was firstly performed on the streamflow data of the Zhidan watershed because it contained missing data. The raw data were preprocessed in Microsoft Excel and imported to SPSS version 23 [43]. The SPSS software has been recommended in several studies [34,44,45,46,47]. Exploratory data analysis (EDA) was performed to check for quality control.

1. Descriptive statistics

This was implemented by graphing the catchment data to draw emphasis on the missing data amounts and the missing data locations by plotting the daily streamflow of the Zhidan watershed. The average monthly streamflow data for each year was also plotted to have a first look at the seasonality apparent in the Zhidan watershed to roughly guess the approximate values for the missing portions of the data due to the knowledge obtained.

To further understand the scope of the missing data problem, descriptive and diagnostic test statistics were conducted on the Zhidan data series. This was done to ascertain the descriptive statistics, missingness ratio, normality, or non-normality of the raw Zhidan streamflow dataset. A significant question pertinent to missing time series data is when the threshold of missingness is beyond a certain critical value (e.g., 5% as proposed by Little et al. [6]), a strong justification for employing a suitable missing data method is presented [6]. The normality or non-normality of the dataset was further explored by drawing the distribution of the dataset.

2. Missingness mechanism and missingness pattern

In addition, we applied Little’s MCAR test [48] to determine the mechanism of missingness (i.e., missing at random (MAR), missing completely at random (MCAR), and missing not at random (MNAR)). The missing data pattern was ascertained by thoroughly investigating the whole dataset to determine whether it has a monotone or general (non-monotone) pattern.

According to Little and Rubin, and van Buuren [1,49], missing data patterns may follow the monotone or general (non-monotone) patterns. In practice, the pattern of missing data is rarely monotone but is often close to monotone [49]. Also, van Buuren [1] states that univariate missing data form a special monotone pattern and significant computational savings are possible if the data are monotone.

3. Decomposition and Auto-correlation

These tests were implemented because the Zhidan streamflow data is a single station dataset. The dataset is classified as a univariate time series with no other additional hydrological variables available. Time series decomposition divides the time series into single component series representing a certain characteristic or pattern such as trends, seasonalities, or irregular components [4].

Also, according to Moritz et al. [4], the idea of measuring autocorrelation is that forecasting (and also imputation) of a time series is conceivable, since the future usually depends on the past, thus high autocorrelation means that the future is strongly correlated to the past, and therefore making autocorrelation an indicator for the ability to generate reliable forecasts and imputations.

Thus, the knowledge of the auto-correlation and decomposition of the dataset influences the imputation methods for missing data reconstruction. Decomposition and auto-correlation were implemented in the R statistical software program [50] using an enhanced version of the Seasonal and Trend decomposition using Loess (STL) (stlplus package) [51,52] which allows for missing data, and autocorrelation function (acf function) [53] where the na.action = na.pass function can be called to handle missing values.

The complete Maduwang dataset was then transformed into a missing dataset by removing data to fit the Zhidan watershed’s missingness to compare the different missing data imputation algorithms. EDA was then repeated on the complete and transformed Maduwang dataset. Little’s test was not applied to the transformed Maduwang dataset since it was made to mimic the missingness of the Zhidan dataset.

#### 2.2.2. Missing Data Imputation

1. Imputation methods

The missing data imputation methodology applied in this study was implemented considering the univariate time series dataset used and the investigation of other mechanisms to expand the missing data reconstruction of univariate time series data.

The mechanisms of performing imputation for univariate time series can be grouped into three categories according to Moritz et al. [4]: univariate algorithms which work with univariate inputs without mostly considering the time series aspect of the data, univariate time series algorithms which also work with univariate inputs but account for the time series characteristics of the data, and multivariate algorithms on lagged data which cannot be applied on univariate data but use the time aspect of the time series to create and add covariates in the form of lags and leads to the input to be able to apply multivariate imputation algorithms.

The univariate time series algorithms and multivariate algorithms on lagged data which account for the time characteristics of the data consider the time aspect of the dataset as an implicit variable.

The time aspect of a time series dataset, especially a univariate time series, although implicit, is a variable nonetheless. Therefore, in this study, we investigated the imputation of univariate time series with multivariate algorithms by taking time as an explicit variable and compare their performances to the well-known univariate missing value imputation algorithms.

We also applied a multivariate algorithm that can use the time characteristics of the dataset to generate and add covariates in the form of polynomials of time to the model to be able to apply multivariate imputation.

The MI mechanism was added to this study because of the different imputed data obtained to account for the uncertainties of the imputation process. The EM algorithm was also investigated due to its provision of severally iterated missing data values based on the distribution of the observed data. However, it does not provide multiply imputed results.

This study, therefore, considers the univariate time series algorithms and multivariate algorithms on lagged data categories for univariate time series imputation and the additional methods to compare the differences in the efficiency of these algorithms on observed field univariate time series dataset with missing values.

The missing data imputation methods applied were selected from the available algorithms in the imputeTS package [10,54] of R developed specifically for missing value imputation of univariate time series, multiple imputation and expectation-maximization (EM) using the SPSS software [43,55] and multiple imputation using the mice [56,57] and Amelia [58,59] packages also in R which mostly focus on missing value imputation of multivariate data.

The imputeTS algorithms represent univariate time series algorithms, the MI and EM mechanisms of SPSS, mice algorithms demonstrate the multivariate algorithms with time as explicit and the Amelia algorithms exhibit multivariate algorithms with time as explicit, multivariate algorithms on lagged data and multivariate algorithms using polynomials of time.

The multiple imputation algorithms applied in this study were of different forms and are presented briefly.

Multiple imputation in SPSS with the Monotone mechanism: This is a non-iterative method that can be implemented only when the data have a monotone pattern of missing values. For each variable in the monotone order, the monotone method fits a univariate (single dependent variable) model using all preceding variables in the model as predictors, then imputes missing values for the variable being fit [55].

Multiple imputation using fully conditional specification (FCS) or Multivariate imputation by chained equations (MICE), the algorithm of the mice package: FCS was introduced in 2006 by van Buuren et al. [60] to refer to a general class of methods that specify imputation models for multivariate data as a set of conditional distributions. FCS imputes multivariate missing data on a variable-by-variable basis and necessitates a specification of an imputation model (univariate imputation model) for each incomplete variable and iteratively generates imputations per variable [1,60,61]. FCS is also known as MICE [62].

Multiple implementation with expectation-maximization with bootstrapping (EMB), the algorithm of the Amelia package: The expectation-maximization with bootstrapping (EMB) algorithm applies the well-known expectation-maximization algorithm on multiple bootstrapped samples of the original incomplete data to draw values of the complete-data parameters.

The algorithm then draws imputed values from each bootstrapped parameter, replacing the missing values with these draws [63]. According to Honaker et al. [58,63], the EMB algorithm performs faster, with larger numbers of variables, and is much easier to use, than various other multiple imputation methods, but produces essentially the same answers.

Table 1 shows the classifications of the algorithms and brief descriptions of the methods used are given.

All multiple imputation algorithms used five (5) imputation (m = 5), and the random forests algorithm applied the ten (10) trees (ntree = 10), which is the default. The default number of trees was utilized since it has been proven to perform identically to hundred (100) trees [57]. The EM algorithm used twenty-five (25) iterations for imputation.

The multiple imputation using the mice package algorithms run also using a monotone sequence of imputation as in the SPSS software because univariate missing data form a special monotone pattern [1].

The dataset used in the imputeTS package was a time series object. At the same time, it was a dataframe with the date variable specified as a date object for the multiple imputations using mice and Amelia in R. This was performed because the imputeTS package requires a time series object to run. The mice and Amelia packages require multivariate data.

The linear regression, Bayesian linear regression, and Amelia algorithms produced some implausible imputations (that is negative streamflow values). Therefore, some bounds (considering the seasonal time period of missing values in the dataset) were added to the model to resolve this shortcoming.

The implementation of the lags and leads argument in Amelia which usually is implemented returned an error as:

*Amelia Error Code: 61*

*There is only 1 column of data after removing the ts, cs and idvars. Cannot impute without adding polytime.*

Therefore, the lags and leads argument in Amelia was executed by adding the polytime arguments. This could mean that Amelia would create and add both covariates of polynomials of time and lags and leads to the imputation model.

2. Imputation Accuracy statistics

The determination of imputation accuracy of the missing data reconstruction methods applied to the transformed Maduwang dataset was executed by evaluating the fit between imputed missing data and observed streamflow at daily scales using known goodness-of-fit methods. Table 2 presents the statistical metric equations for evaluating goodness-of-fit between observed streamflow *(*$Q_{o})$ and the predicted (imputed) streamflow $\left(Q_{p}\right)$ which were employed in this study. A detailed description of these metrics can be found in Yapo et al., Moriasi et al., Dembélé and Zwart, Thiemig et al. and Beck et al. [77,78,79,80,81].

Moriasi et al. [78] suggested that RMSE and MAE close to 0 are considered ideal. Also, Singh et al. [82] mentioned that RMSE values less than half of the standard deviation of the observations (referred to here as Std. Dev.) may be considered low. However, Moriasi et al. [78] recommends that RMSE of less than or equal to 70% of the standard deviation of the observed runoff is acceptable (that is RSR ≤ 0.7 is acceptable). Also, the best value of PBIAS is 0 but PBIAS of ±25% for streamflow is acceptable [78].

The imputation accuracy measurements mentioned were performed for the whole extent of the data set to obtain the efficiency of using the various missing data reconstruction schemes. This error measurement of the whole extent of the data is referred to in this study as Total Error Measurement (TEM).

In addition, to assess the closer precision of the different imputation algorithms applied to the real-world field data with missing values in this study, we performed what we refer to as the Localized Error Measurement (LEM), where the error statistics were calculated again for the specific missing data portion in the data, since the missing values were observed to be centered between 2003 and 2005 of the Zhidan dataset. It should be noted that LEM would be effective mostly in cases where the missing data are localized at specific points in the data.

Therefore, TEM would be adequate in cases where the missing values occur vastly spread within the dataset as obtained when missing values are randomly introduced into complete datasets as seen in much of the literature on missing data imputation.

LEM was implemented to “zoom in” to the intrinsic differences between the imputed data and the complete data (for the reference dataset) and observe the changes in the error statistics (especially the bias). This process is especially important for hydrological parameters, since the imputed data might be further used for modeling and any introduced bias before the modelling process could result in unacceptable or unrealistic results. Because the imputed data would be utilized for further analysis, this study considers ±10% or lower as the allowable bias for imputation.

It should be noted that, because the streamflow dataset from the Zhidan watershed are actual observed field data with missing data whose exact values are unknown, the imputation accuracy statistics mentioned above cannot be applied because there is no complete dataset to compare the imputation algorithms to. Therefore, other metrics apart from prediction error statistics are required to observe the efficacy of the various imputation methods to reconstruct the missing data.

Therefore, in this study, metrics such as the mean, standard deviation, variances of the complete, transformed, and imputed values of the Maduwang dataset were additionally calculated and compared. This was performed to determine which imputation algorithm best fits the real-world field dataset (Zhidan catchment) whose missing values are unknown.

**Figure 2:**
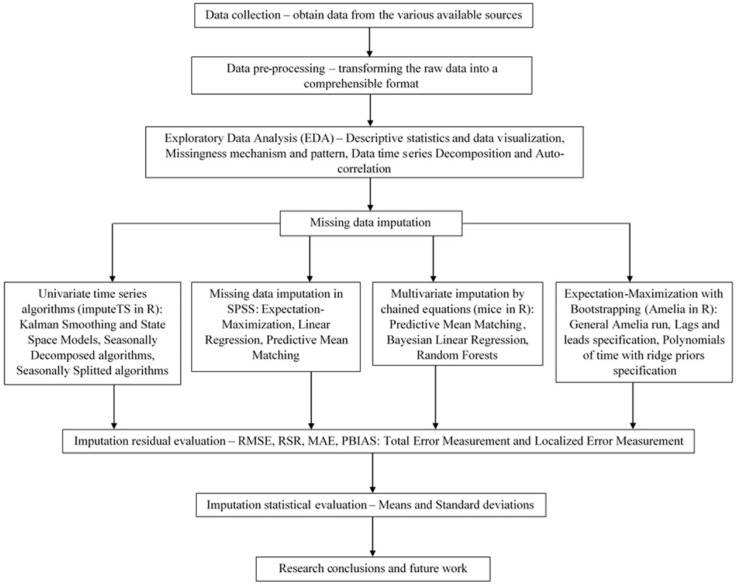
The research design process implemented in this study.

The research block diagram is illustrated in Figure 2 for easy clarification of the research design process implemented in this work.

## 3. Results

### 3.1. EDA Results

**Figure 3:**
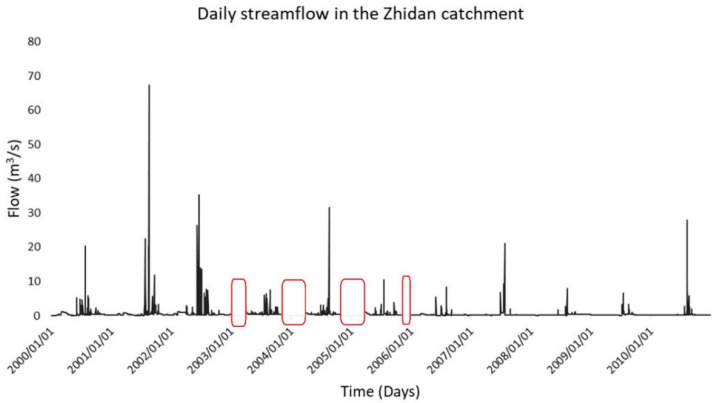
The results of the plot of the raw Zhidan streamflow data series based on the exploratory data analysis (EDA).

The plot of the daily streamflow of the Zhidan watershed showing the missing data portions in red is presented in Figure 2. A look at the streamflow dataset of the Zhidan watershed demonstrated that the missing data areas were concentrated in January to March and November to December of the years 2003 to 2005. These are represented in Figure 3.

The results of the descriptive statistics, normality and MCAR test results of Zhidan catchment data are further displayed in Table 3 and Figure S1.

In Table 3, the analysis of the missing data in the Zhidan streamflow time series dataset showed 412 missing values out of 4018 total values (equivalent to a percentage of approximately 10.3% missingness), exceeding the critical missingness threshold of 5%, and therefore conventional methods of data infilling such as averaging and interpolation are not recommended. We also assessed convergence by reporting the means and standard deviation.

In addition, the skewness and kurtosis results from Table 3, showed that the data is largely positively skewed with a large kurtosis proving its non-normality. The data also series satisfies the non-normality assumption from results of the Kolmogorov–Smirnov test (*p* = 0.0) and the Shapiro–Wilk test (*p* = 0.0) (Table 3) respectively.

Figure S1 also presents the non-normality of the Zhidan dataset by showing a highly positively skewed distribution, suggesting that missing data schemes which do not assume normal distribution would obtain better results. The results of Little’s test on the Zhidan dataset are also depicted in Table 3, where the significance value is less than 0.05 and we can conclude that the data are not missing completely at random but might be MAR or MNAR instead.

Table 4 and Table 5 present the descriptive statistics and normality test while Figure S2 illustrates the distribution for the complete and transformed Maduwang dataset respectively.

Table 4 and Table 5 show that some variation occurs in the data when the complete Maduwang dataset is transformed to contain missing data as seen in the differences in mean, standard deviation, skewness, and kurtosis. However, we noticed that the non-normal distribution of the dataset remains intact as perceived from the significance values of the normality tests (Kolmogorov–Smirnov test (*p* = 0.0) and the Shapiro–Wilk test (*p* = 0.0)). This observation is also confirmed in Figure S2, where we see both the complete and transformed Maduwang dataset having the same distribution.

Comparing Figure S1 to Figure S2, which means comparing the distribution of the Zhidan dataset to the Maduwang dataset, shows that both are highly positively skewed having the same distribution, therefore, eliminating the issue of data distribution bias in comparing the missing data reconstruction methods in this particular study.

**Figure 4:**
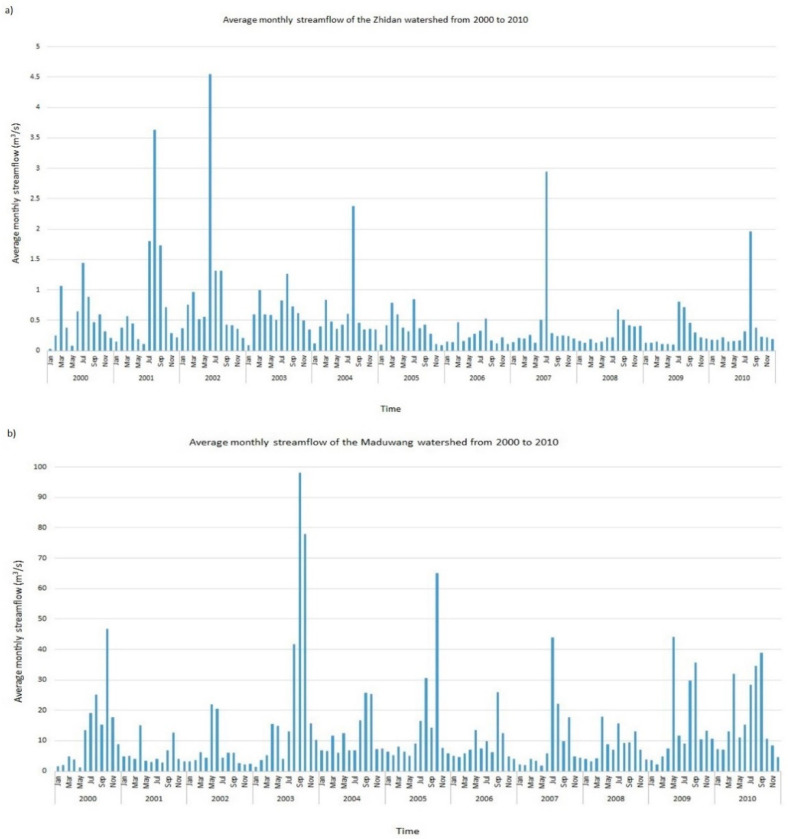
Plot of the average monthly streamflow of the (**a**) Zhidan and (**b**) complete Maduwang datasets.

The plot of the average monthly streamflow of the Zhidan dataset and the complete Maduwang dataset is also illustrated in Figure 4. This plot was performed to have a rough view of the seasonality of the data in the watershed and to be able to assess the efficiency of the imputation algorithms applied in this study by gaining knowledge and understanding the characteristics of the datasets based on the characteristics of the watersheds.

In statistics, adding a data point above the mean, or removing a data point below the mean, increases the mean. Similarly, removing a data point above the mean, or adding a data point below the mean, decreases it. In light of this, observing Figure 4b, it can be seen that the general average monthly streamflow values of January to March and November to December (even in the years of 2003 to 2005) are below the overall mean of the Maduwang dataset (which is 12.56 as shown in Table 4).

Removing the data points below the mean during the transformation process increases the newly obtained mean. This can be observed in the mean of the transformed Maduwang dataset (seen as 13.16 in Table 5), and using the past and future data characteristics of the Zhidan dataset, the same pattern is seen of the average monthly streamflow values of January to March and November to December.

We can safely conclude that the mean of the Zhidan dataset with missing values is an increased mean resulting from having some data points below the overall mean of the dataset missing. Although the data points of January to March and November to December are mostly below the mean of the datasets of both watersheds, their spread is closer to the mean than for the data points of the wetter seasons (typically July to October). Removing these data points in the transformation process (in the case of the Maduwang dataset) or having them missing (in the case of the Zhidan watershed) increases the spread of the data points and therefore increases the standard deviation.

This can be observed by comparing the standard deviations of the complete Maduwang dataset (Table 4) and the transformed Maduwang dataset (Table 5). This knowledge greatly influenced the choice of missing data imputation algorithms envisioned to be the best for filling the missing streamflow values for the watershed.

Figure S3 represents the decomposed time series of the complete and transformed Maduwang dataset. Figure S3b shows a pre-imputed decomposition of the transformed Maduwang dataset (imputed regions shown in red) performed automatically by the stlplus package by filling the series gap with the nearest neighbors [32] in its attempt to handle the missing data to produce the time series decomposition.

The differences in the decomposed complete and transformed Maduwang dataset are most noticeable in the trend plot, which shows the areas of pre-imputed data in the trend decomposition forming straight lines (Figure S3b) instead of the more curved nature of the complete dataset (Figure S3a). This means that imputation methods can also be compared in this way to ascertain their accuracy in mimicking the trend of the complete dataset. The Maduwang dataset is seen to possess both seasonality and trend. The trend is somewhat increasing although not pronounced.

Figure S4 presents the autocorrelation results of the complete and transformed Maduwang dataset. By default, the acf function in R does not allow for missing values, and therefore when the na.action = na.pass function is used, the covariances are computed from the complete cases [83], that is, list-wise deletion.

From Figure S4, the autocorrelation of the complete and transformed Maduwang dataset are the same. However, this in no way implies that it would be the same in all cases when the acf function is used with missing data. This is because the results from the acf function depend completely on the observed data without any pre-imputation, and therefore the results might be biased if the missing data proportion is fairly large or if the covariance after dealing with the missing data do not reflect the actual autocorrelation of the data without missing data. The Maduwang dataset shows a strong positive autocorrelation.

Figure S5 displays the decomposed time series and autocorrelation results of the Zhidan watershed. The stlplus package pre-imputed the series gap (shown in red), and the acf function applied complete case analysis to deal with the missing data.

The decomposed time series of the Zhidan dataset shows seasonality and a very distinct decreasing trend. We also observed a positive autocorrelation in the data with more statistically significant autocorrelation and some minor statistically insignificant autocorrelation.

### 3.2. Imputation Results

The imputation accuracy statistics of the different missing data reconstruction algorithms applied in this study are presented in Table 6.

In terms of time and computational power consumption, the structural time series method of the Kalman smoothing algorithm and the Kalman method of the seasonally decomposed algorithm were slow and computationally intensive. All other algorithms, especially the multiple imputation mechanisms, converge quickly without much strain on the computing system.

The results of the imputation accuracy statistics in Table 6 demonstrate quite low RMSE for all the applied algorithms across the total error measurement (TEM) and the localized error measurement (LEM). This is shown because the RSR values for all the measured statistics are less than 0.7, as recommended by Moriasi et al. [78], with the highest obtained RSR values in this study being approximately 0.4.

Considering the imputeTS results, the structural time series method of the Kalman smoothing algorithm outperformed the ARIMA state-space representation method of the same algorithm in all the measured error metrics, with the most notable being the PBIAS statistic where the structural time series method presented no bias (0%) with the TEM and a 0.1% overestimation with the LEM, whereas the ARIMA state-space representation method showed underestimating the missing values with both error measurements (3.6% with TEM and 9.7% with LEM).

The interpolation method of the seasonally decomposed algorithm outperformed the Kalman method of the same algorithm in the RMSE (and so RSR) and MAE error metrics. However, the Kalman method demonstrated no bias at all in estimating the missing values, whereas the interpolation method presented minimal underestimation of the missing values across both error measurements (0.1% with TEM and 0.3% with LEM).

The opposite effect occurred in the application of the seasonally splitted algorithm. The Kalman method outperforms the interpolation method in the RMSE (and so RSR) and MAE error metrics, but the interpolation method shows less percentage bias in the estimation of the missing values across both the TEM and LEM, although both methods underestimate the missing values.

Another observation was made of the imputeTS algorithms in Table 6 where, regardless of the better performance of interpolation method of the seasonally decomposed algorithm in the RMSE, RSR, and MAE than all the other imputeTS algorithms, its percentage bias is worse than that of the structural time series method of the Kalman smoothing algorithm and the Kalman method of the seasonally decomposed algorithm across both the TEM and LEM.

Moreover, while the ARIMA state-space representation method of the Kalman smoothing algorithm and the interpolation method of the seasonally splitted algorithm have the same RMSE and RSR values, the latter performs better in the MAE and PBIAS error metrics across both error measurements. Therefore, a general overview of the performance of the imputeTS package algorithms shows the seasonally decomposed algorithm with the interpolation method having the best RMSE, RSR, and MAE results, while the seasonally decomposed algorithm with the Kalman method exhibiting the best bias results across both the TEM and LEM.

The results obtained from the application of the SPSS software show the PMM method of the Monotone multiple imputation mechanism performing the best among all the other schemes both in the TEM and LEM, with some minor underestimation bias (0.1% with TEM and 0.2% with LEM). The EM imputation algorithm follows having satisfactory results across all the error statistics in the TEM and LEM. Still, it obtained a 12% overestimation bias with the LEM, above the allowed bias in this study. The LR method of the Monotone multiple imputation mechanism was noted to struggle with obtaining low bias in both the TEM and LEM albeit the satisfactory scores in RMSE, RSR, and MAE metrics.

The RF method of the MICE algorithm generated the best results among all the applied MICE multiple imputation mechanisms with some slight overestimation bias of 0.3% with the TEM and 0.9% with the LEM, followed by the PMM method, which similarly produced acceptable RMSE, RSR, and MAE results but obtained overestimation biases of 2.8% with the TEM and 7.5% with the LEM.

The BLR method of the MICE multiple imputation algorithm also achieved satisfactory RMSE, RSR, and MAE results but resulted in high overestimating of the bias with both error measurements, especially with the LEM, where we observe a 31.9% overestimation of the missing values.

Amelia offers the EMB multiple imputation method. All of the applied methods are observed to generate acceptable RMSE, RSR, and MAE results with both the TEM and LEM, with the lags and leads method having the best results among the EMB methods. However, we note that all of the methods result in significant overestimation of the missing values across both the TEM and LEM, with the Time as explicit obtaining as high as a 50% overestimation of the missing values and with the LEM far above the allowable 10% bias in this study.

A comparison of all the results in Table 6 indicates overestimation bias with almost all of the multiple imputation algorithms and underestimation bias with the single imputation mechanisms.

The exceptions are the PMM method of the Monotone multiple imputation mechanism implemented in SPSS which produced underestimation of the missing values, and the EM algorithm, also performed in the SPSS software, which resulted in overestimation of the missing values, although it is not a multiple imputation method. The RMSE and MAE values more than doubled to almost tripling in the percentage bias statistics from the TEM to the LEM. Table 6 further demonstrates the differences in the results generated by each algorithm and method used.

For example:

1. The PMM method of the Monotone multiple imputation mechanism in SPSS surpassed the PMM method of the MICE algorithm in all of the error metrics across the TEM and the LEM. However, both use the same predictive mean matching method of missing data imputation, with the Monotone PMM in SPSS underestimating the missing values. At the same time, the PMM of MICE overestimates the missing values.
2. Although the interpolation method of the seasonally decomposed algorithm outperforms all of the applied methods in RMSE, RSR, and MAE, it is overtaken in terms of the bias by the Kalman method of the same algorithm, the structural time series of the Kalman smoothing algorithm across both the TEM and LEM, and the PMM method of the Monotone multiple imputation mechanism in SPSS with the LEM while having the same underestimation bias with the TEM.
3. The PMM method of the Monotone multiple imputation mechanism in SPSS, although having a better RMSE value, possesses the same RSR value and is outdone in MAE and percentage bias across the TEM and LEM by the structural time series of the Kalman smoothing algorithm.
4. Similarly, the PMM method of the Monotone multiple imputation mechanism in SPSS, although having better RMSE and RSR values than the Kalman method of the seasonally decomposed algorithm, shows the same MAE value and is outdone in the estimation percentage bias by the latter method across both the TEM and LEM.
5. The RF method of the MICE algorithm overtakes the ARIMA state-space representation method of the Kalman smoothing algorithm, all of the methods of the seasonally splitted algorithm in MAE and bias results across all of the measurements regardless of its worse RMSE and RSR results, and overestimates the missing values, while the others underestimate the missing values.
6. The PMM method of the MICE algorithm and the Kalman method of the seasonally splitted algorithm possess similar and opposite bias estimation of the missing values, with the PMM overestimating while the Kalman underestimates, although the Kalman method displays better RMSE, RSR, and MAE results.
7. The BLR method of the MICE algorithm also outperforms the LR method of the Monotone multiple imputation mechanism in SPSS and all of the methods of the EMB algorithm in MAE and percentage bias across both the TEM and LEM, although the LR method and the EMB algorithms display better RMSE and RSR values.

The additional statistical metrics for the comparison of imputation algorithms are presented in Table 7 and Table 8.

For the statistical metrics of comparison of the imputation algorithms for the Maduwang dataset shown in Table 7, it is easier to detect the algorithms that performed satisfactorily in terms of the means and standard deviations. We note that the Kalman method of the seasonally decomposed algorithm possesses the same mean and standard deviation as the complete dataset, but with differences in their variance. This is because, although the spread of the values from the mean are similar, the values themselves are not the same.

One is the complete dataset of the Maduwang watershed and the other contains imputed missing values. The structural time series method of the Kalman smoothing algorithm has a higher mean but a lower standard deviation than the complete dataset.

The interpolation method of the seasonally decomposed algorithm has the same standard deviation as the complete dataset, albeit it has a lower mean than the complete dataset. However, the PMM method of the Monotone multiple imputation mechanism in SPSS was observed to have a greater standard deviation than the complete dataset, although it has a lower mean.

The RF method of the MICE algorithm is viewed to have a higher mean and standard deviation than the complete dataset. The differences in the means of the above-mentioned algorithms and methods to the complete Maduwang dataset can be considered insignificant. The ARIMA state-space representation method of the Kalman smoothing algorithm has a higher standard deviation than the complete dataset although it also has a lower mean, and the PMM method of the MICE algorithm is seen to have a greater mean and standard deviation that the complete dataset. The differences in the means, however, of the ARIMA state-space representation method of the Kalman smoothing algorithm and the PMM method of the MICE algorithm with the complete dataset could be argued to be slightly significant.

The EM algorithm in SPSS produced a mean greater than the complete dataset but less than the mean of the transformed dataset and a standard deviation lower than both complete and transformed datasets.

A thorough inspection of the imputed missing value results of the EM algorithm showed that they were filled with values similar or near to the mean of the complete and transformed dataset and thus this is the reason for the smaller standard deviation than both the complete and transformed datasets. The LR method of the Monotone multiple imputation mechanism in SPSS, the BLR method of the MICE algorithm, and all of the methods of the EMB algorithm produced higher means than both the complete and transformed datasets. These algorithms generated higher means than the transformed Maduwang dataset when the expectation was to achieve the opposite, due to the data characteristics. Although below that of the transformed dataset, their standard deviations cannot be considered at this point because of the significant disparity in their means in comparison to the transformed dataset. Thus, the differences in the means of these algorithms in addition to the EM algorithm with the complete dataset are considered highly significant.

Table 8 shows that all of the methods of the seasonally splitted algorithm did not impute one missing value. This caused their means to be slightly significantly below the mean of the complete dataset. The cause of this discrepancy is uncertain, and further work could be conducted to ascertain the reasons for, and solutions to, this issue.

For the statistical metrics of the Zhidan dataset in Table 8, some inferences from statistical knowledge, knowledge of the dataset characteristics in Figure 4, and the Maduwang dataset in Table 7 were employed.

1. The mean of the original Zhidan dataset containing missing data is a greater mean than the mean of the entire dataset without missing data. Therefore, the algorithms which resulted in lower means than the original dataset provide better missing data imputation methods for this real-world dataset.
2. The seasonally splitted algorithm did not impute one missing value also of the Zhidan watershed in Table 8, similar to the case displayed in Table 7. Therefore, it can be rightly assumed that the means produced by this algorithm would be below the actual mean of the Zhidan dataset without missing values.

Therefore, with the Zhidan watershed, the range of the expected mean of the new dataset without missing values was observed to between 0.507 and 0.523, and the missing data imputations which produced such results could be considered good methods to fill in the missing data of this real-world dataset.

It was noted that the Kalman smoothing, seasonally decomposed algorithms with all their methods, the PMM method of the Monotone multiple imputation mechanism in SPSS, and the RF method of the MICE algorithm fit these characteristics for the expected mean The LR method of the Monotone multiple imputation in SPSS, PMM, and BLR methods of the MICE algorithm and all of the methods of the EMB algorithm do not.

## 4. Discussion

The localized error measurement (LEM) showed a tripling of the imputation accuracy error measurements compared to the total error measurement (TEM) imputation accuracy of the different imputation algorithms employed in this study, because the LEM method showed the actual differences in values between the imputed missing data and the complete dataset, although the results of the TEM appeared to be accurate for most of the imputation algorithms. This was an eye-opening observation, in that, in this case where there was about 10.3% of the data missing, we have the difference in the complete and transformed dataset being about an 89.7% match in the application of the TEM, even in the face of different imputed values, and therefore would expect an imputation accuracy corresponding to this match. However, when we zoomed in to only 10.3% of the complete dataset and the imputed dataset using the LEM, the variance between the complete dataset and the imputed missing values was extremely highlighted, especially in terms of the bias of estimation. Therefore, this LEM is recommended when the missing values are concentrated at specific and easily differentiable portions of the dataset to be imputed.

The univariate missing value algorithms (Kalman smoothing, seasonally decomposed, and seasonally splitted) performed satisfactorily because the datasets employed in this study are typical univariate time series datasets, which is the main data which these algorithms could cater to. Nevertheless, we observed that multiple imputation algorithms such as the PMM method of both the Monotone multiple imputation in SPSS and MICE algorithm and the RF method of the MICE algorithm, which are commonly accustomed to multivariate data, performed as well as these univariate missing value algorithms and in some cases better, especially with the bias, which shows that these multiple imputation algorithms could be considered as highly sophisticated.

The predictive mean matching (PMM) method of both the Monotone multiple imputation in SPSS and MICE algorithm was noted to perform well because PMM utilizes knowledge of the actual observed values surrounding the missing data to build the imputation model. And therefore, for data such as the ones utilized in this study, this process greatly comes in handy in improving the imputation accuracy of the missing values.

The PMM method of the Monotone multiple imputation in SPSS outdid the PMM method of the MICE algorithm, although both apply the same predictive mean matching method of missing data imputation. This is because of the differences in the rendering of the calculation of the multiply imputed values. The MICE algorithm is an iterative multiple imputation algorithm [62], whereas the Monotone mechanism of SPSS is non-iterative [55]. The MICE algorithm generates the missing values by repeating the imputation sequences using the Gibbs sampler for each imputation [57,62]. MICE applies five (5) iterations for each of the five (5) imputations, while SPSS performs no iterations for each of the five (5) imputations. This could mean that the more multifaceted algorithm is better adapted to complex scenarios, such that simpler problems create some struggles. Therefore, because the datasets in this study contained two variables, with one variable as date data, the simpler imputation method performed best.

Similarly, the random forests (RF) method of the MICE algorithm also performed well when the other MI methods struggled because the imputation model of the RF method is slightly more complex than the others, in that random forests behave in a similar fashion to neural networks, with the tree splitting and creation of nodes process, and thus its learning of the data is also better, resulting in better imputation accuracy results.

The biased mean result obtained by the EM algorithm was because the algorithm fitted the dataset to a normal distribution where the mean value of this distribution possesses the highest probability density in the entire population.

The LR method of the Monotone multiple imputation in SPSS, the BLR method of the MICE algorithm, and all of the methods of the EMB algorithm, even though they produced acceptable RMSE, RSR, and MAE results, struggled with achieving better imputation accuracy in terms of bias because of the randomness of their search for missing values in the imputation models. Moreover, these algorithms even necessitated the addition of bounds to their imputation models since they produced negative streamflow values.

Furthermore, thorough inspection of the imputed Zhidan and Maduwang missing values by the LR method of the Monotone multiple imputation in SPSS, BLR method of the MICE algorithm, and all of the methods of the EMB algorithm showed that the variability in the imputed values of the Zhidan watershed was smaller than that of the Maduwang watershed because of the smaller range of the Zhidan dataset. For example, the Maduwang dataset has minimum and maximum streamflow values of 0.22 m^3^/s and 575 m^3^/s respectively (see Table 4), making the range of the data equal to 574.78 m^3^/s, while the Zhidan dataset has minimum and maximum streamflow values of 0.023 m^3^/s and 67.4 m^3^/s respectively (see Table 3), with a range of 67.38 m^3^/s. The imputation algorithms that were given bounds for the imputation model, and therefore performed better with smaller range because of the constraints in the degrees of freedom in obtaining the missing values. In light of this observation, it can be perceived that the range and bounds of the dataset also play a vital role in influencing imputation accuracy.

Moreover, by discerning the algorithms and methods whose means are insignificant or slightly significant in Table 7 and comparing them to the imputation accuracy measures of the Maduwang dataset in Table 6, it can be noticed that these algorithms performed best among the implemented imputation methods with both the TEM and LEM, and were also seen to fit the expected mean criteria of the Zhidan dataset (Table 8). Therefore, in the case of real-world datasets such as the Zhidan dataset, these algorithms provide good and efficient methods for missing value imputation.

The actual mean, standard deviation, and variance of the Zhidan streamflow dataset are unknown and, therefore, to ascertain the best imputation algorithm applied requires some additional effort. This is where knowledge of the climatic characteristics of the catchment (the wet and dry periods) and the positions of the missing values are highly significant. Also, some knowledge of the flood event periods in the watershed would be of supplementary benefit. This knowledge allows the imputer to guess better the possible range of values of the missing data in order to choose the best missing data algorithm, especially when the imputer is only applying the TEM for imputation accuracy. From the Zhidan dataset, it was observed that the missing data was localized between January to March and November to December of the years 2003 to 2005. These months represent the driest periods of the catchment. There were also no flood events recorded in the Zhidan watershed within these periods, and therefore the expectance of outliers is greatly reduced. In this case, the missing value imputation algorithms that take the dataset’s seasonality and/or the surrounding observed values of the missing data into consideration would perform best, and the imputation methods that produce more random imputed missing values would perform the worse. Therefore, it can be concluded that missing value imputation of streamflow time series data or, more generally, hydroclimatic time series data in the real-world do not solely depend on the imputation algorithms implemented but also on extensive study of the particular dataset to be imputed.

## 5. Conclusions

This paper contributes to our understanding and highlights recent developments in filling missing data, particularly in geographical areas that often suffer from inadequate hydrological data by tackling questions such as: (1) can multiple imputation methods be used with accuracy on univariate time series data and how do they compare with univariate time series algorithms; and (2) how do we handle missing data in real-world data and is imputation alone enough? Finally, the following conclusions were made from the results and observations attained.

Univariate missing value algorithms are specially developed to handle univariate time series data and provide satisfactory results depending on the time decomposition characteristics of the data. However, the univariate missing value algorithms which provide the best results are usually time and computationally intensive.

The time aspect of a time series dataset, especially a univariate time series, although implicit, is a variable nonetheless. Therefore, even though these algorithms are accustomed to multivariate data, multiple imputation algorithms which take the surrounding observed values into consideration, like predictive mean matching (PMM), or which can understand the characteristics of the data like random forests (RF) provide similar results to the univariate missing data algorithms and in some cases perform better without the added time and computational downsides when time is taken as an explicit variable.

The localized error measurement (LEM) recommended in this study would provide deeper insights into the actual bias between the imputed missing values and the complete dataset. This is especially useful when the missing data are concentrated at specific portions of the dataset or when very large gaps of missingness occur.

In the case of the real-world Zhidan streamflow dataset, it was determined that to ascertain the best imputation algorithm applied requires some additional knowledge of the catchment characteristics such as climatic characteristics and flood event periods because of outliers to make sure that the missing values are replaced with plausible values. Therefore, proper handling of missing values of real-world streamflow or hydroclimatic datasets depends on imputing and extensive study of the particular dataset to be imputed.

More work is required to investigate the details of the failure of the seasonally splitted algorithm to impute one missing data value.

The proposed methodology implemented in this study necessitates further verification with the application of several other datasets from different catchments to further expand and improve the understanding of missing data imputation techniques.

In addition, much research is also needed on missing data imputation of real-world datasets, necessitating the introduction and/or development of more metrics for finding the efficiency of imputation algorithms in applying these datasets, because error measurements such as RMSE, RSR, MAE, and PBIAS, among others, become inapplicable with real-world data since they require complete data for comparison.

Further research would include obtaining more hydrological parameters of the watersheds to explore the changes in performance accuracy of the multivariate missing data imputation algorithms, especially in their handling of the time characteristics of hydroclimatic data.

## Figures and Tables

**Table 1:** Classifications of the algorithms and brief descriptions of the methods used in this study.

Imputation Method	Method	Multiple Imputation	Description of Imputation Methods and Methods
imputeTS (univariate time series algorithms)			
Kalman Smoothing and State Space Models (na_kalman)	Structural Model & Kalman Smoothing (StructTS)	No	It implements Kalman Smoothing on structural time series models or on the state space representation of an ARIMA model for imputation [54]. Details on the Kalman Filtering and State Space Models can be found in Harvey [64], Welch and Bishop [65], and Grewal and Andrews [66].
	ARIMA State Space Representation & Kalman Smoothing (auto.arima)	No	
Seasonally Decomposed Missing Value Imputation (na_seadec)	Imputation by Interpolation algorithm after decomposition (interpolation)	No	This method, as a preprocessing step, firstly decomposes the data and removes the seasonal component from the time series. Then performs imputation on the trend and irregular components and afterwards adds the seasonal component again [9,54].
	Imputation by Kalman Smoothing and State Space Models after decomposition (kalman)	No	
Seasonally Splitted Missing Value Imputation (na_seasplit)	Imputation by Interpolation algorithm after split (interpolation)	No	This method, also as a preprocessing step, initially splits the times series into seasons and afterwards performs imputation separately for each of the seasons [54].
	Imputation by Kalman Smoothing and State Space Models after split (kalman)	No	
SPSS (multivariate algorithms with time as explicit)			
Expectation-Maximization (EM)		No	EM is an iterative algorithm to find maximum likelihood estimation problem for missing data. Likelihood-based approaches define a model for the observed data and the inferences are based on the likelihood or posterior distribution under the posited model [1]. The major idea of this algorithm is to calculate the values of missing variables according to the initial parameters (means, covariance) and observed data (E step). Then update the initial parameters according to the complete data set that has been calculated (M step) and repeat the two steps until convergence. Little and Rubin, and Schafer provide extensive review of the theory of the EM algorithm [46,67].
Monotone	Linear Regression (LR)	Yes	Multiple imputation using linear regression generates imputations by building a model from observed data and predicting the missing values from the fitted model using the spread around the fitted linear regression line of y given x, as fitted on the observed data [1,57]. Here, the analysis is performed by point estimates to find the single best value around the regression line [68].
	Predictive Mean Matching (PMM)	Yes	Predictive mean matching is a multiple imputation mechanism where the missing values are drawn from the observed data such that it always finds values that have been actually observed in the data so that it is close to the predicted mean by using an implicit model and the nearest-neighbor together to calculate the values [1,69]. Imputations are restricted to the observed values, and hence PMM can maintain non-linear relations when the structural part of the imputation model is inaccurate [62], so they are realistic and, therefore, imputations outside the observed data range will not occur, thus avoiding problems of meaningless imputations [1].
mice (multivariate algorithms with time as explicit)			
Fully conditional specification (FCS) or Multivariate imputation by chained equations (MICE)	PMM (mice.impute.pmm)	Yes	As above
	Bayesian Linear Regression (BLR) (mice.impute.norm)	Yes	Multiple imputation using Bayesian linear regression [70] is much like linear regression. However, the imputation is done within the context of Bayesian inference where the missing values are drawn from a Bayesian posterior predictive distribution for the observed data [68,71]. Thus BLR seeks to find out the posterior distribution for the model parameters rather than finding a single best value [68].
	Random Forests (RF) (mice.impute.rf)	Yes	Random forests [72] use machine learning by combining many regression trees (for continuous variables) or classification trees (for discrete variables) into an ensemble by drawing several bootstrap samples (a random sample of predictors as the covariates before each node is split) [1,73,74,75]. RF consists of iteratively training a random forest on observed values for imputing the missing values [25]. Thus Random forest applies a set of observed input–output training data to create predictions of the mean output for new input data [76].
Amelia (multivariate algorithms with time as explicit, multivariate algorithms on lagged data and multivariate algorithms using polynomials of time)			
Expectation-Maximization with Bootstrapping (EMB)	General Amelia run (Time as explicit)	Yes	This was run as the general form of Amelia without the specification of the time series argument but with the date variable as a date object in R
	Lags and leads (lags and leads method)	Yes	A way of handling time-series information in Amelia is to include lags and leads of certain variables into the imputation model. Lags are variables that take the value of another variable in the previous time period while leads take the value of another variable in the next time period [4,58]. Amelia then adds covariates of lags and leads of the specified variable to the imputation model.
	Time series polynomials with ridge priors (ts and polytime method)	Yes	Another way Amelia allows for the consideration of time series data is with the use of the ts and polytime arguments. Using the ts and polytime arguments, Amelia can develop a general model of patterns within variables across time by creating a sequence of polynomials of the time index of time up to the user defined k-th order, (k ≤ 3). With this input, Amelia will add covariates to the model that are equivalent to time and its polynomials and these covariates will help better impute the missing values [58].

**Table 2:** Commonly used statistical metric for evaluating the two methods.

Statistic Metric	Equation	Values Range	Perfect Score
Root Mean Square Error (RMSE)	$RMSE=\sqrt{\frac{\sum_{i=1}^{n}{\left({Q_{o}}_{i}-{Q_{p}}_{i}\right)}^{2}}{n}}$	0–∞	0
Ratio of RMSE to the standard deviation of the observations (RSR)	$RSR=\frac{RMSE}{Std.\;Dev.}=\frac{\left[\sqrt{\sum_{i=1}^{n}{\left({Q_{o}}_{i}-{Q_{p}}_{i}\right)}^{2}}\right]}{\left[\sqrt{\sum_{i=1}^{n}{\left({Q_{o}}_{i}-\bar{Q_{o}}\right)}^{2}}\right]}$	0–1	0
Mean Absolute Error (MAE)	$MAE=\frac{\sum_{i=1}^{n}\left|{Q_{o}}_{i}-{Q_{p}}_{i}\right|}{n}$	0–∞	0
Percent of bias (PBIAS)	$PBIAS=\frac{\sum_{i=1}^{n}({Q_{o}}_{i}-{Q_{p}}_{i})}{\sum_{i=1}^{n}({Q_{o}}_{i})}\times 100\%$	0–100%	0

where *n* represents the number of cases of each station; ${Q_{o}}_{i}$ and ${Q_{p}}_{i}$ represents daily observed streamflow and the predicted (imputed) streamflow at time *i* respectively. $\bar{Q_{o}}$ = mean observed values and $\bar{Q_{p}}$ = mean predicted (imputed) values.

**Table 3:** Descriptive statistics, Normality and MCAR test results of Zhidan catchment data.

		Descriptive Statistics			
$\mathrm{Observed}\;(Z_{OBS})$		$\mathrm{Missing}\;(Z_{MIS})$		Total	
N	%	N	Percent (%)	N	%
3606	89.7%	412	10.3%	4018	100.0%
Mean (SE)	Std. Dev.	Minimum	Maximum	Skewness	Kurtosis
0.523 (0.032)	1.906	0.023	67.40	18.836	510.412
Test of Normality					
Kolmogorov-Smirnov ^a^			Shapiro-Wilk		
Statistic	df	Sig	Statistic	df	Sig.
0.397	3606	0.000	0.153	3606	0.000
Little’s MCAR Test					
Chi-Square		df		Sig.	
50.239		1		0.000	

N denotes number of counts of the Zhidan data time series obtained. SE denotes standard error. ^a^ denotes Lilliefors Significance Correction.

**Table 4:** Descriptive statistics and Normality test results of the complete Maduwang catchment data.

$\mathrm{Observed}\;(M_{OBS})$		$\mathrm{Missing}\;(M_{MIS})$		Total	
N	%	N	Percent (%)	N	%
4018	100%	0	0%	4018	100.0%
Mean (SE)	Std. Dev.	Minimum	Maximum	Skewness	Kurtosis
12.557 (0.462)	29.310	0.22	575.00	8.543	101.410
Test of Normality					
Kolmogorov-Smirnov ^a^			Shapiro-Wilk		
Statistic	df	Sig	Statistic	df	Sig.
0.337	4018	0.000	0.316	4018	0.000

N denotes number of counts of the Maduwang data time series obtained. SE denotes standard error. ^a^ denotes Lilliefors Significance Correction.

**Table 5:** Descriptive statistics and Normality test results of the transformed Maduwang catchment data.

$\mathrm{Observed}\;(Z_{OBS})$		$\mathrm{Missing}\;(Z_{MIS})$		Total	
N	%	N	Percent (%)	N	%
3606	89.7%	412	10.3%	4018	100.0%
Mean (SE)	Std. Dev.	Minimum	Maximum	Skewness	Kurtosis
13.161(0.514)	30.857	0.22	575.00	8.102	91.096
Test of Normality					
Kolmogorov-Smirnov ^a^			Shapiro-Wilk		
Statistic	df	Sig	Statistic	df	Sig.
0.337	3606	0.000	0.328	3606	0.000

N denotes number of counts of the Maduwang data time series obtained. SE denote standard error. ^a^. Lilliefors Significance Correction.

**Table 6:** Imputation accuracy statistics of the different missing data reconstruction algorithms.

		Total Error Measurement (TEM)				Localized Error Measurement (LEM)			
Imputation Method	Method	RMSE (m^3^/s)	RSR	MAE (m^3^/s)	PBIAS	RMSE (m^3^/s)	RSR	MAE (m^3^/s)	PBIAS (%)
imputeTS									
na_kalman	StructTS	0.541	0.018	0.089	0	1.036	0.025	0.326	0.1
	auto.arima	1.690	0.058	0.458	−3.6	3.237	0.079	1.680	−9.7
na_seadec	interpolation	0.465	0.016	0.081	−0.1	0.890	0.022	0.298	−0.3
	kalman	0.575	0.020	0.095	0	1.100	0.027	0.347	0
na_seasplit	interpolation	1.690	0.058	0.407	−2.6	3.237	0.079	1.494	−6.9
	kalman	1.655	0.056	0.401	−2.8	3.170	0.077	1.471	−7.5
SPSS									
EM		2.126	0.073	0.623	4.5	4.071	0.099	2.283	12
Monotone	LR	7.827	0.267	2.280	18.1	14.986	0.364	8.357	48.2
	PMM	0.537	0.018	0.095	−0.1	1.028	0.025	0.347	−0.2
mice									
FCS or MICE	PMM	5.172	0.176	0.616	2.8	9.902	0.241	2.257	7.5
	BLR	8.352	0.285	1.946	12	15.991	0.389	7.134	31.9
	RF	1.727	0.059	0.227	0.3	3.307	0.080	0.834	0.9
Amelia									
EMB	Time as explicit	8.056	0.275	2.362	18.8	15.425	0.375	8.658	50
	Lags and leads	7.185	0.245	2.057	16.3	13.757	0.334	7.543	43.5
	ts and polytime	7.830	0.267	2.300	18.3	14.993	0.364	8.431	48.8

**Table 7:** Statistical metrics for comparison of imputation algorithms for the Maduwang dataset.

Data		N	Mean	Std. Dev.	Variance
Complete		4018	12.557	29.310	859.096
Transformed		3606	13.161	30.857	952.128
na_kalman	StructTS	4018	12.560	29.309	858.993
	auto.arima	4018	12.099	29.409	864.899
na_seadec	interpolation	4018	12.544	29.310	859.104
	kalman	4018	12.557	29.310	859.047
na_seasplit	interpolation	4017	12.232	29.368	862.486
	kalman	4017	12.205	29.374	862.827
EM		4018	13.121	29.232	854.497
Monotone	LR	4018	14.830	29.793	887.619
	PMM	4018	12.548	29.312	859.197
FCS or MICE	PMM	4018	12.910	29.712	882.828
	BLR	4018	14.060	30.144	908.644
	RF	4018	12.594	29.341	860.913
EMB	Time as explicit	4018	14.915	29.841	890.514
	Lags and leads	4018	14.607	29.700	882.105
	ts and polytime	4018	14.856	29.802	888.174

**Table 8:** Statistical metrics for comparison of imputation algorithms for the Zhidan dataset.

Data		N	Mean	Std. Dev.	Variance
Original dataset		3606	0.523	1.907	3.635
na_kalman	StructTS	4018	0.513	1.807	3.266
	auto.arima	4018	0.512	1.809	3.272
na_seadec	interpolation	4018	0.514	1.810	3.276
	kalman	4018	0.515	1.808	3.270
na_seasplit	interpolation	4017	0.507	1.809	3.271
	kalman	4017	0.506	1.808	3.269
EM		4018	0.530	1.806	3.263
Monotone	LR	4018	0.653	1.855	3.442
	PMM	4018	0.513	1.810	3.276
FCS or MICE	PMM	4018	0.527	1.839	3.383
	BLR	4018	0.593	1.862	3.469
	RF	4018	0.511	1.810	3.275
EMB	Time as explicit	4018	0.643	1.849	3.419
	Lags and leads	4018	0.636	1.845	3.404
	ts and polytime	4018	0.652	1.854	3.438

## Data Availability

The data utilized in this study are available at the local hydrological bureaus of the Zhidan and Maduwang watersheds. Any interested person(s) could contact these organizations for the data.

## Supplementary Materials

The following are available online at https://www.mdpi.com/article/10.3390/ijerph18168375/s1, Figure S1. Distribution curve of the streamflow dataset of the Zhidan watershed. Figure S2. Distribution curve of the (a) complete and (b) transformed streamflow dataset of the Maduwang watershed. Figure S3. Time series decomposition of (a) complete and (b) transformed Maduwang dataset. Figure S4. Autocorrelation of (a) complete and (b) transformed Maduwang dataset. Figure S5. Results of (a) time series decomposition and (b) autocorrelation of the Zhidan dataset.
