# Supplementary material for: Comparison of Missing Data Infilling Mechanisms for Recovering a Real-World Single Station Streamflow Observation
Source: Int J Environ Res Public Health. 2021 Aug 7;18(16):8375. doi: 10.3390/ijerph18168375 (PMC8394992; doi:10.3390/ijerph18168375)
Supplement: Supplementary file 1 [file ijerph-18-08375-s001.zip › ijerph-1301151-supplementary.pdf]

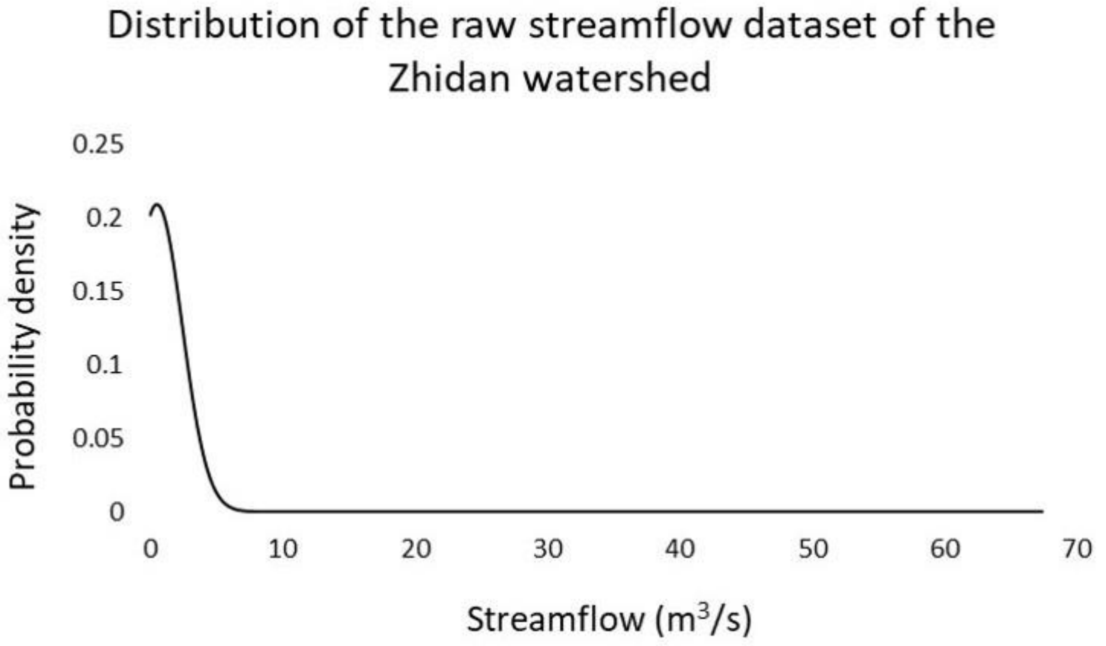

Figure S1. Distribution curve of the streamflow dataset of the Zhidan watershed.

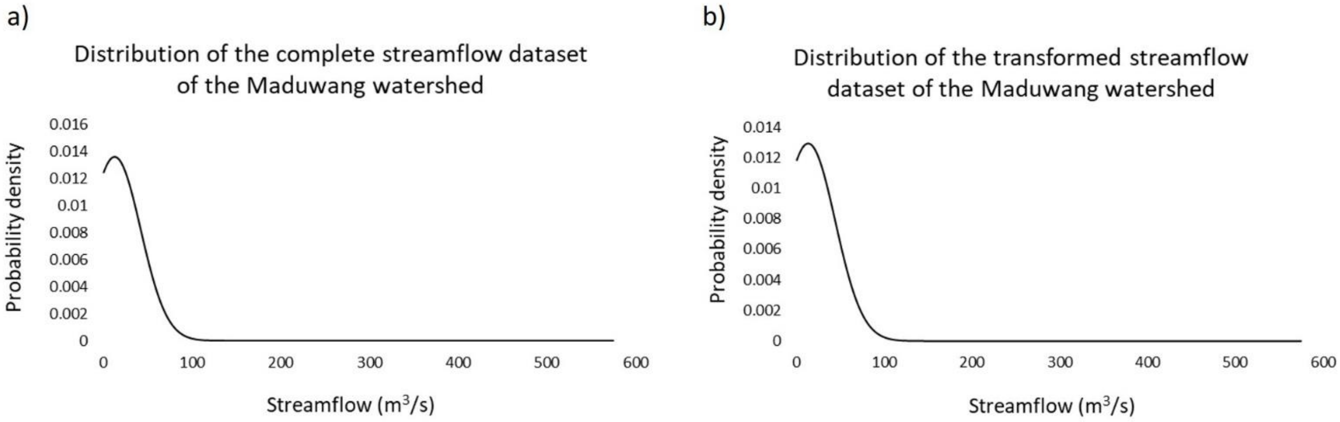

Figure S2. Distribution curve of the (**a**) complete and (**b**) transformed streamflow dataset of the Maduwang watershed.

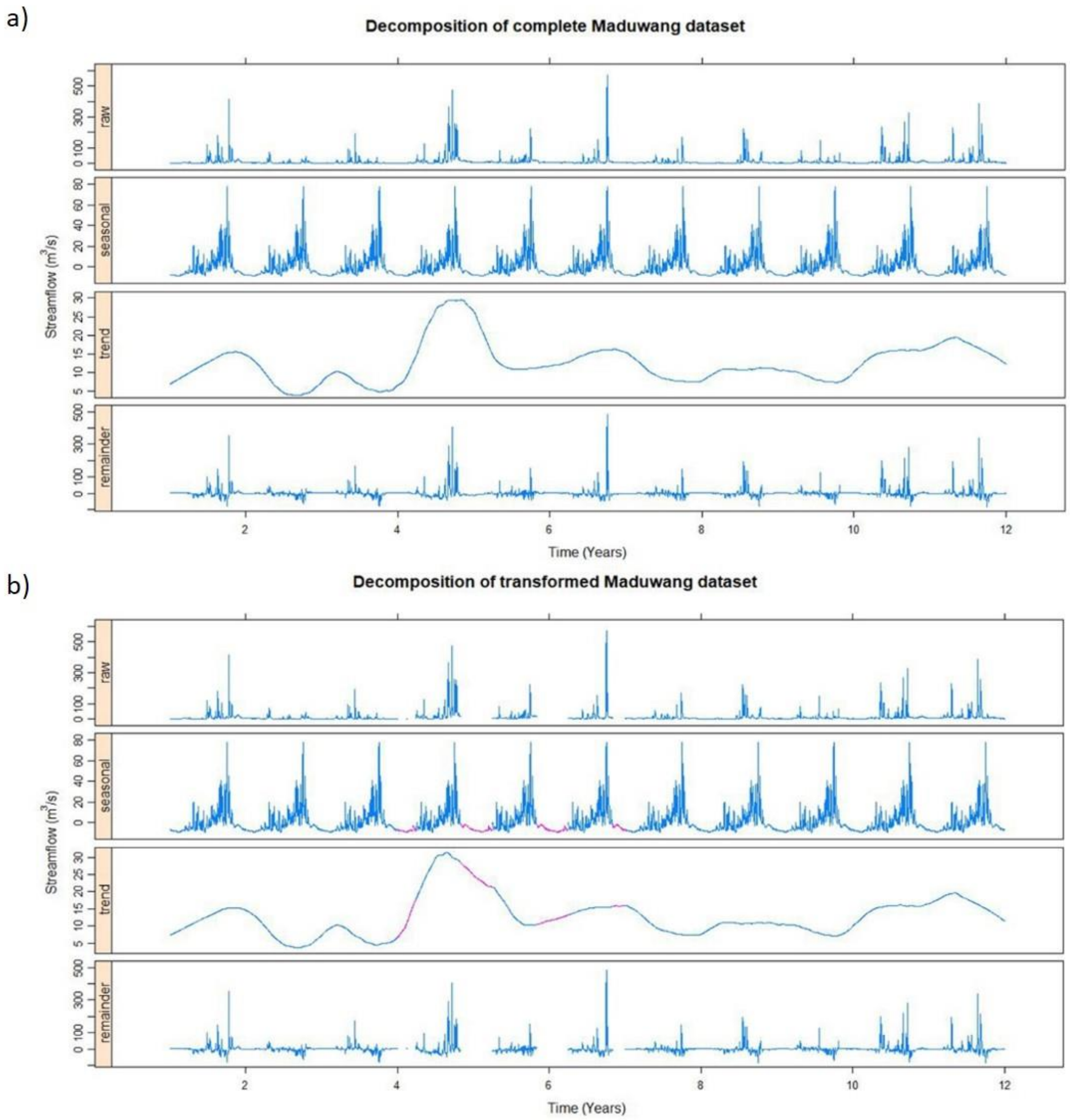

Figure S3. Time series decomposition of (**a**) complete and (**b**) transformed Maduwang dataset.

a)

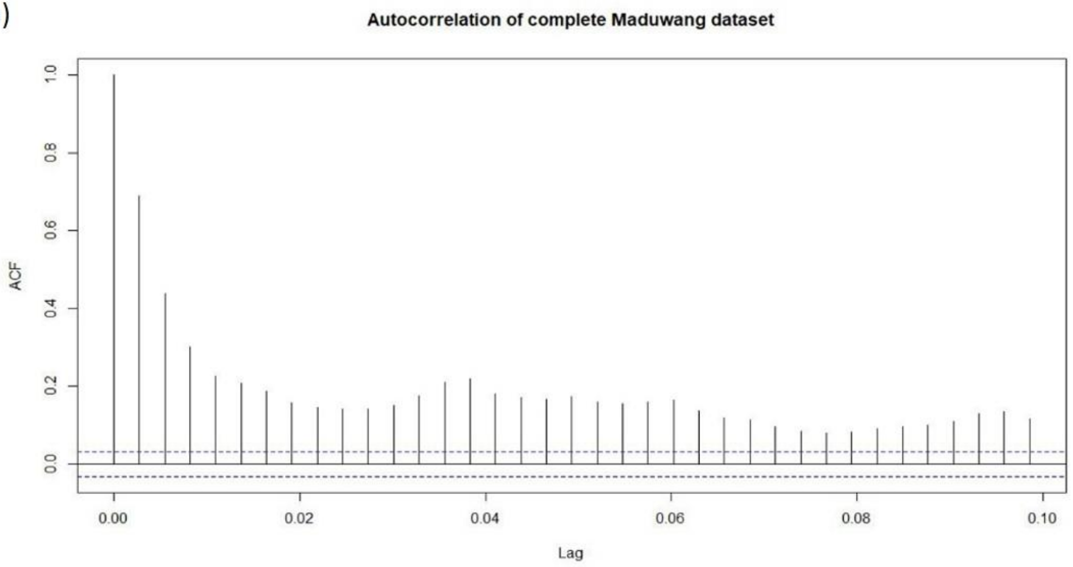

b)

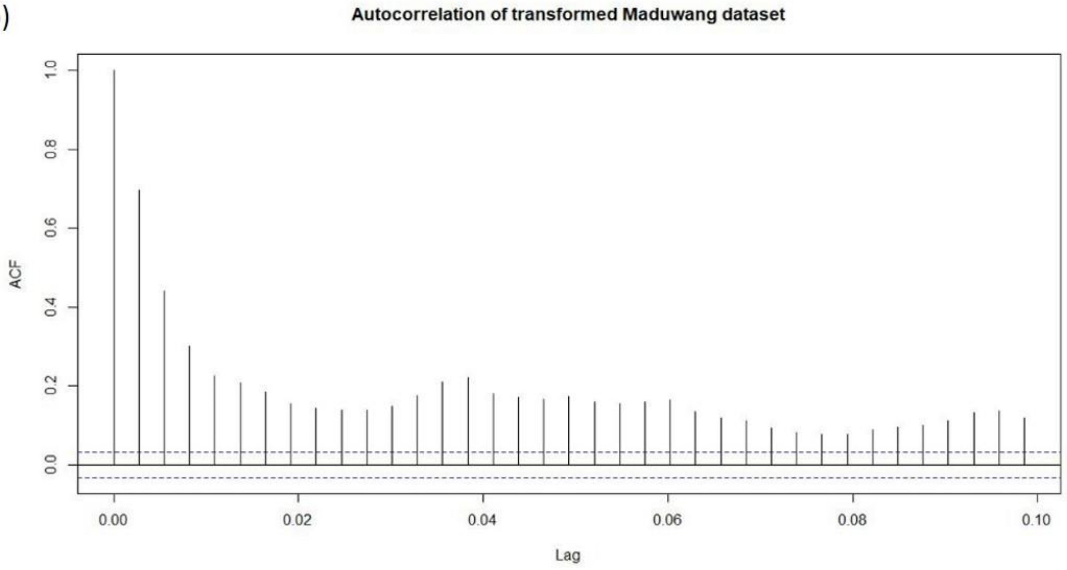

Figure S4. Autocorrelation of (**a**) complete and (**b**) transformed Maduwang dataset.

a)

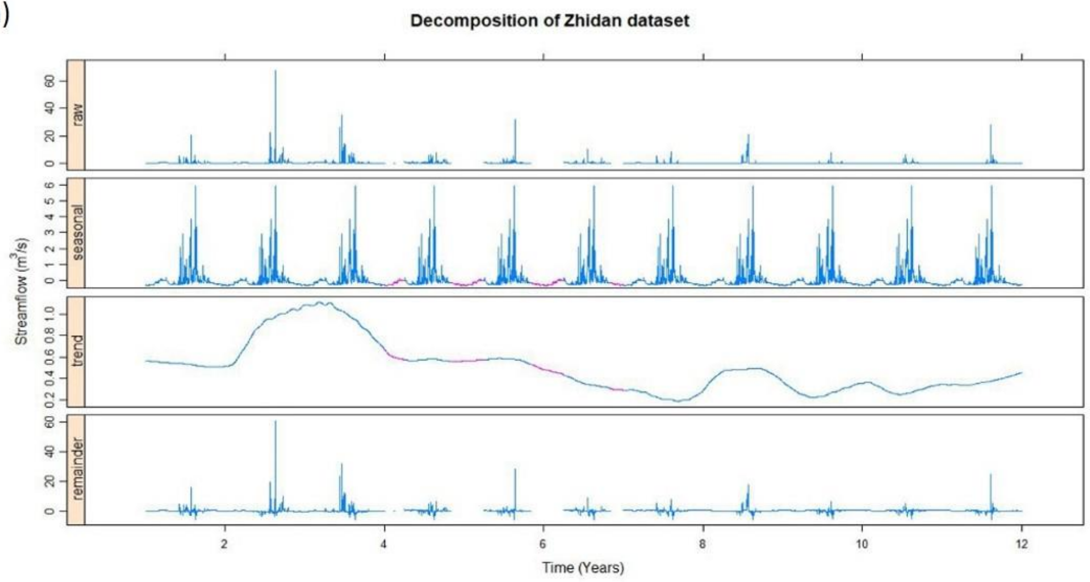

b)

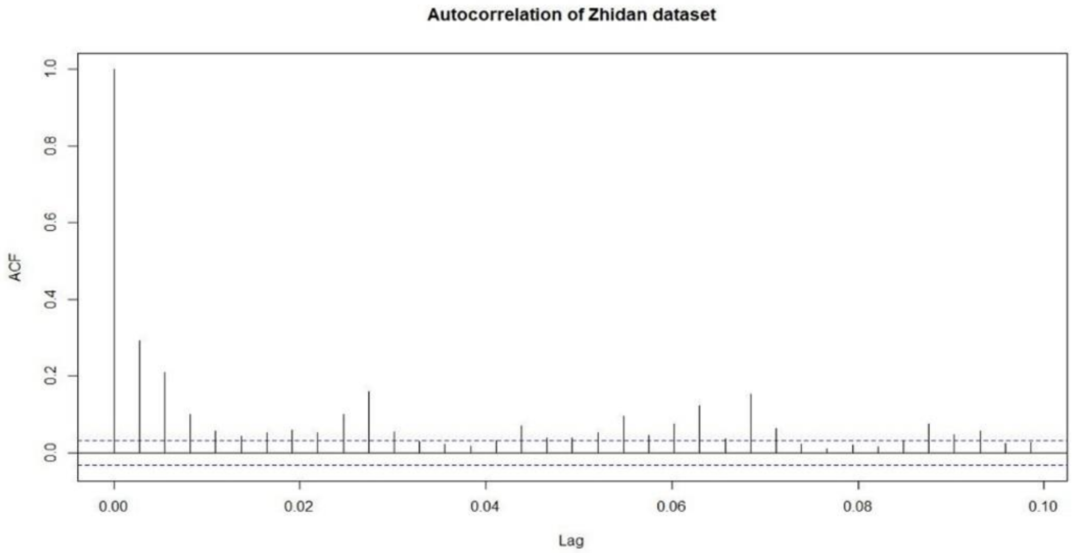

Figure S5. Results of (**a**) time series decomposition and (**b**) autocorrelation of the Zhidan dataset.
